# Sex differences in discriminating between cues predicting threat and safety

**DOI:** 10.1016/j.nlm.2016.07.014

**Published:** 2016-09

**Authors:** Harriet L.L. Day, Molly M. Reed, Carl W. Stevenson

**Affiliations:** School of Biosciences, University of Nottingham, Sutton Bonington Campus, Loughborough LE12 5RD, UK

**Keywords:** Discrimination, Fear, Generalization, Post-traumatic stress disorder, Safety, Threat

## Abstract

Post-traumatic stress disorder (PTSD) is more prevalent in women than men. PTSD is characterized by overgeneralization of fear to innocuous stimuli and involves impaired inhibition of learned fear by cues that predict safety. While evidence indicates that learned fear inhibition through extinction differs in males and females, less is known about sex differences in fear discrimination and safety learning. Here we examined auditory fear discrimination in male and female rats. In Experiment 1A, rats underwent 1–3 days of discrimination training consisting of one tone predicting threat (CS+; presented with footshock) and another tone predicting safety (CS−; presented alone). Females, but not males, discriminated between the CS+ and CS− after one day of training. After 2–3 days of training, however, males discriminated whereas females generalized between the CS+ and CS−. In Experiment 1B, females showed enhanced anxiety-like behaviour and locomotor activity in the open field, although these results were unlikely to explain the sex differences in fear discrimination. In Experiment 2, we found no differences in shock sensitivity between males and females. In Experiment 3, males and females again discriminated and generalized, respectively, after three days of training. Moreover, fear generalization in females resulted from impaired safety learning, as shown by a retardation test. Whereas subsequent fear conditioning to the previous CS− retarded learning in males, females showed no such retardation. These results suggest that, while females show fear discrimination with limited training, they show fear generalization with extended training due to impaired safety learning.

## Highlights

• We investigated sex differences in auditory fear discrimination in rats.
• Males that received extended discrimination training showed fear discrimination.
• Females discriminated after limited training and generalized after extended training.
• Generalization with extended training in females involved impaired safety signaling.

## 1 Introduction

It is now well established that there are marked sex differences in the prevalence of post-traumatic stress disorder (PTSD). For example, women are twice as likely to be diagnosed with PTSD compared to men (Lebron-Milad and Milad, 2012, McLean et al., 2011). The reasons for this marked discrepancy remain unclear and are thought to be multi-factorial in nature (Catuzzi and Beck, 2014, McLean and Anderson, 2009). Understanding the neurobiological basis of sex differences in the vulnerability to develop PTSD has been severely hindered by the paucity of preclinical studies that have investigated fear learning and memory processing in female animals (Lebron-Milad & Milad, 2012). Including females in such translational research will provide valuable insight on the contributing factors underlying sex differences in PTSD susceptibility, which may lead to the development of more effective interventions or treatments.

More recent animal studies have begun to redress this imbalance by examining sex differences in the regulation of learned fear using translationally relevant behavioral paradigms. A hallmark feature of PTSD is the impaired inhibition of fear. A growing body of evidence indicates that PTSD is associated with deficits in the extinction of learned fear (Graham and Milad, 2011, Jovanovic and Norrholm, 2011). In this form of fear inhibition, repeated presentations of a discrete conditioned stimulus (CS) or prolonged exposure to a conditioned context in the absence of the unconditioned stimulus (US) decreases fear responding to the CS or context. Recent human studies have demonstrated that women show impaired extinction memory recall, which is influenced by estrogen (Glover et al., 2012, Graham and Milad, 2013, Milad et al., 2006, Milad et al., 2010, Zeidan et al., 2011). Animal research has also shown impaired fear extinction processing in females and these studies are beginning to elucidate the underpinning neural circuit, neurochemical, and endocrine mechanisms (Baker-Andresen et al., 2013, Baran et al., 2010, Fenton et al., in press, Fenton et al., 2014, Matsuda et al., 2015, Milad et al., 2009, Rey et al., 2014).

Another characteristic of PTSD is the overgeneralization of fear to innocuous stimuli or contexts. Fear discrimination and generalization can be investigated using comparable behavioral paradigms in humans and animals, where one cue (CS+) predicts threat through its association with the US and the other cue (CS−) signals safety by predicting that the US will not occur (Dunsmoor & Paz, 2015). Fear discrimination can be viewed as a form of learned fear inhibition by the safety cue and emerging evidence has led to the conceptualization of fear generalization in PTSD as a deficit in fear inhibition due to impaired safety signaling (Christianson et al., 2012, Jovanovic and Norrholm, 2011). Although a failure to discriminate between cues predicting threat and safety has been proposed as a biomarker of PTSD, little is known about sex differences in fear discrimination. Recent human and animal studies have demonstrated impaired contextual fear discrimination in females (Lonsdorf et al., 2015, Lynch et al., 2013, Reppucci et al., 2013). However, sex differences in fear discrimination involving discrete cues, and the role of altered safety signaling in mediating these differences, are poorly understood.

In the present study we investigated sex differences in auditory fear discrimination in male and female rats by examining learned fear behaviour (i.e. freezing) in response to two distinct tones predictive of threat or safety. As previous studies have shown sex differences in the rates of learning using various aversive conditioning paradigms (Dalla & Shors, 2009), we examined the effects of both limited and extended discrimination training on later retrieval. We also determined if any sex differences in freezing observed during discrimination retrieval were attributable to non-specific effects on anxiety-like behaviour, locomotor activity, and/or nociception. Finally, we investigated whether sex differences in fear discrimination with extended training involve altered safety signaling by using a retardation test to examine the inhibitory properties of the safety cue during subsequent fear conditioning (Christianson et al., 2012, Rescorla, 1969, Sangha et al., 2013).

## 2 Material and methods

### 2.1 Animals

Experiments were performed on young adult male and naturally cycling female Lister hooded rats (Charles River, UK). Rats were group housed (4–5/cage) by sex in individually ventilated cages on a 12-h light/dark cycle (lights on at 8:00) with access to food and water *ad libitum*. All experiments were conducted with internal ethical approval and in accordance with the Animals (Scientific Procedures) Act 1986, UK. All behavioral testing occurred during the rats’ light cycle.

### 2.2 Experiment 1A: Auditory fear discrimination training and retrieval testing

**Fig. 1:**
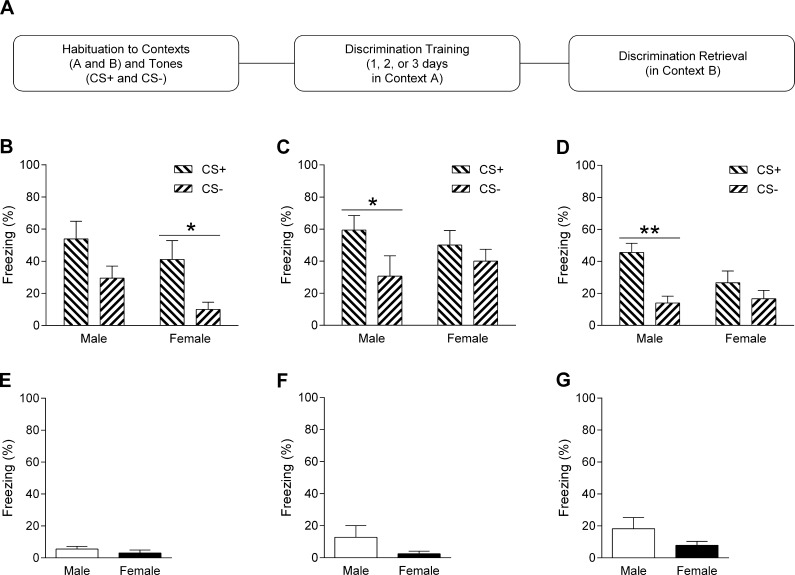
Sex differences in auditory fear discrimination depend on the extent of training received. (A) Schematic representation of the fear discrimination paradigm used. (B) Freezing in response to CS+ and CS− presentation during discrimination retrieval testing after one day of training. Males showed no significant difference in freezing between the CS+ and CS−, whereas freezing was increased during CS+ compared to CS− presentation in females (^*^P < 0.05). (C and D) Freezing to the CS+ and CS− during retrieval testing after two (C) or three (D) days of training. Freezing was increased to the CS+ compared to the CS− in males (^*^P < 0.05, ^**^P < 0.01), while freezing during CS+ and CS− presentation did not differ in females. (E–G) Freezing before CS+ and CS− presentations during retrieval testing after one (E), two (F), or three (G) days of training. There were no significant differences in freezing between any of the males and females.

The rats underwent auditory fear discrimination training and retrieval testing using two chambers that have been described elsewhere (Stevenson, Spicer, Mason, & Marsden, 2009). On the first day rats were habituated for 10 min each to contexts A and B, which had distinct visual (black and white stripes or spots on two walls), olfactory (40% ethanol or 40% methanol), and tactile (metal floor bars or white Perspex floor) cues. During habituation the rats were also presented with 2 and 9 kHz tones (two presentations of each; 30 s, 80 dB, 2 min inter-trial interval (ITI)) in both contexts. From the next day, separate cohorts of males and females underwent one, two, or three days of fear discrimination training in context A, resulting in six separate groups of rats undergoing behavioral testing. This consisted of five presentations of one tone (CS+; 30 s, 80 dB, 2 min ITI) paired with footshock (0.5 s, 0.5 mA, ending at tone offset) and five presentations of a different tone alone (CS−; 30 s, 80 dB, 2 min ITI). The CS+ and CS− tones used were 2 or 9 kHz and fully counterbalanced between rats. One day after the last day of discrimination training rats received two presentations each of the CS+ and CS− in context B to test discrimination retrieval (Fig. 1A). Tone and footshock delivery were controlled automatically by a computer running MED-PC IV software (Med Associates, VT). Rats were tested at approximately the same time of day on each day and behaviour was recorded with a digital camera for later data analysis. The chambers were cleaned with 40% ethanol (context A) or 40% methanol (context B) between each testing session.

### 2.3 Experiment 1B: Open field testing

The rats that underwent two days of discrimination training in Experiment 1A were also tested in the open field to examine sex differences in anxiety-like behaviour and locomotor activity using an apparatus described elsewhere (Heath et al., 2015). Open field testing occurred the week before fear discrimination testing. Rats were placed in the open field for 10 min and behaviour was digitally recorded during testing for later data analysis. The floor of the open field was cleaned with 40% methanol between each session.

### 2.4 Experiment 2: Shock sensitivity testing

A separate cohort of rats was used to examine sex differences in shock sensitivity as described elsewhere (Heath et al., 2015). Rats were placed in the chambers and after two min received 10 unsignalled footshocks (0.5 s, 1 min ITI) of increasing intensity (0.05–0.5 mA in 0.05 mA increments). Behaviour during the test was digitally recorded for later data analysis and the chambers were cleaned with 40% ethanol between each session.

### 2.5 Experiment 3: Auditory fear discrimination and retardation testing

A separate cohort of rats underwent auditory fear discrimination followed by retardation testing to examine sex differences in safety signaling by the CS−. Half of the rats were habituated to the two contexts and tones, underwent three days of discrimination training in context A, and were tested for discrimination retrieval in context B as in Experiment 1A above. The day after discrimination retrieval testing the rats were habituated for 10 min to context C, which had distinct visual (complete darkness) and olfactory (1% acetic acid) cues. The next day the rats were subjected to auditory fear conditioning in context C using the previous CS− as the conditioned cue. This consisted of five presentations of the tone (30 s, 2 min ITI; 2 or 9 kHz, fully counterbalanced) paired with footshock (0.5 s, 0.5 mA, ending at tone offset). The following day the rats received two presentations of the cue alone in context B to test fear retrieval. Control rats were subjected to these same procedures except that no shocks were presented during discrimination training, which served to pre-expose the controls to the same number of tones before auditory fear conditioning (Fig. 4A). In the retardation test, if fear discrimination results in the CS− acting as a safety signal then later fear conditioning to that CS− is impaired (or retarded) in relation to controls conditioned to the pre-exposed cue; this, in turn, results in reduced learned fear responding compared to the pre-exposed controls at fear retrieval test. Rats were tested at approximately the same time of day on each day and behaviour was digitally recorded for later data analysis. The chambers were cleaned with 40% ethanol (context A), 40% methanol (context B), or 1% acetic acid (context C) between each session.

### 2.6 Data analysis

In Experiment 1A, freezing (i.e. absence of movement except relating to respiration) in response to CS+ and CS− presentations during discrimination retrieval testing was quantified. Freezing was scored manually by 2–3 trained observers. The observers scored freezing blind to the CS type and one was blind to the sex of the rats. Freezing was determined at 3 s intervals during tone presentation. The cumulative duration of freezing was then calculated and expressed as a percentage of the 30 s tone. The mean percentage of freezing during each of the two tones (CS+ and CS−) was calculated and used in the statistical analysis. Sex differences in freezing during CS+ and CS− presentation were analyzed separately in the different groups of rats that underwent one, two or three days of discrimination training using two-way analysis of variance (ANOVA), with sex and CS type as between- and within-subject factors, respectively. Direct comparisons between freezing during CS+ and CS− presentation in males and females that underwent one, two or three days of discrimination training were also conducted separately using independent paired *t*-tests (Keeley et al., 2015, Lynch et al., 2013). Contextual fear was inferred from freezing during the 2 min period before tone presentations, which was scored as above. Sex differences in contextual fear were analyzed separately in the different groups that underwent one, two or three days of discrimination training using independent paired t-tests.

In Experiment 1B, behaviour in the open field was analyzed using Ethovision software (Noldus, Netherlands). The time spent in, latency to enter, and frequency of entries into the center were quantified as indices of anxiety-like behaviour, while the horizontal distance moved throughout the whole open field during testing was quantified as an index of locomotor activity (Heath et al., 2015, Prut and Belzung, 2003, Stevenson et al., 2009). Sex differences in the anxiety-like behavioral measures were analyzed using two-way ANOVA, with sex and measure as between- and within-subject factors, respectively. Sex differences in locomotor activity were examined separately by analyzing the horizontal distance moved using an unpaired *t*-test.

In Experiment 2, the threshold current needed to elicit ‘flinch’ responses and audible vocalizations during shock sensitivity testing were scored manually (Heath et al., 2015). Sex differences in flinch and vocalization responses were analyzed using two-way ANOVA, with sex and response type as between- and within-subject factors, respectively.

In Experiment 3, freezing in response to CS+ and CS− presentations during discrimination retrieval testing was determined and sex differences were then analyzed using two-way ANOVA as in Experiment 1A above. In the subsequent retardation test, freezing in response to cue presentations during fear retrieval testing was quantified and the mean percentage of freezing during the two cues was calculated and used in the statistical analysis. Sex differences in freezing during cue presentation between rats subjected previously to fear discrimination training or cue pre-exposure were analyzed using two-way ANOVA, with sex and cue history as between-subject factors. Direct comparisons between freezing in discrimination trained and cue pre-exposed controls in males and females were also conducted separately using independent unpaired *t*-tests.

All data are presented as the mean plus the standard error of the mean. All post hoc comparisons were conducted using the Bonferroni’s test where indicated. The level of significance for all comparisons was set at P < 0.05.

## 3 Results

### 3.1 Experiment 1A: Sex differences in fear discrimination depend on the extent of training received

The fear discrimination paradigm used in Experiment 1A is outlined in Fig. 1A. Freezing in response to CS+ and CS− presentation during fear discrimination retrieval testing after one, two, or three days of fear discrimination training is shown in Fig. 1B–D. In males (n = 9) and females (n = 9) subjected to one day of training (Fig. 1B), the two-way ANOVA revealed a significant main effect of CS type (F_(1,16)_ = 10.86, P = 0.005) but no main effect of sex (F_(1,16)_ = 2.71, P = 0.12) or sex × CS type interaction (F_(1,16)_ = 0.16, P = 0.68). Despite the lack of significant interaction, we were interested in examining differences in freezing during CS+ and CS− presentation in males and females. Direct comparisons were therefore conducted using independent unpaired *t*-tests. Males showed more freezing in response to CS+ compared to CS− presentation but this did not reach statistical significance (t_(8)_ = 2.13, P = 0.066). However, females did show a significant increase in freezing during CS+ compared to CS− presentation (t_(8)_ = 2.52, P = 0.036).

In males (n = 8) and females (n = 8) subjected to two days of training (Fig. 1C), the two-way ANOVA again revealed a significant main effect of CS type (F_(1,14)_ = 12.21, P = 0.004) but no main effect of sex (F_(1,14)_ = 0.0, P > 0.99) or sex × CS type interaction (F_(1,14)_ = 2.86, P = 0.11). Despite there being no significant interaction, direct comparisons were conducted to examine differences in freezing in response to the CS+ and CS− in each sex. Males again showed more freezing during CS+ compared to CS− presentation and this difference reached statistical significance (t_(7)_ = 3.37, P = 0.01). In contrast, females showed no difference in freezing in response to the CS+ and CS− (t_(7)_ = 1.41, P = 0.20).

In males (n = 10) and females (n = 9) subjected to three days of training (Fig. 1D), the two-way ANOVA revealed a significant main effect of CS type (F_(1,17)_ = 24.66, P = 0.0003) and a significant sex × CS type interaction (F_(1,17)_ = 5.55, P = 0.031) but no main effect of sex (F_(1,17)_ = 1.47, P = 0.24). Post hoc analysis indicated that males showed significantly increased freezing during CS+ compared to CS− presentation (P < 0.001), while females showed no such difference (P > 0.05). This was confirmed by the direct comparison analysis, which showed that freezing was significantly increased in response to the CS+ compared to the CS− in males (t_(9)_ = 4.31, P = 0.002) but not females (t_(8)_ = 1.95, P = 0.087). Taken together, these results suggest that extended training resulted in better fear discrimination in males, while females showed fear discrimination with limited training and fear generalization with extended training.

Freezing before CS+ and CS− presentations during fear discrimination retrieval testing after one, two, or three days of fear discrimination training is shown in Fig. 1E–G. Although males showed more freezing than females, this did not reach significance in the rats that underwent one (t_(16)_ = 1.01, P = 0.33; Fig. 1E), two (t_(14)_ = 1.40, P = 0.18; Fig. 1F), or three (t_(17)_ = 1.34, P = 0.20; Fig. 1G) days of discrimination training. This finding suggests that there were no sex differences in contextual fear before testing auditory fear discrimination retrieval.

### 3.2 Experiment 1B: Females exhibit enhanced anxiety-like behaviour and locomotor activity in the open field

**Fig. 2:**
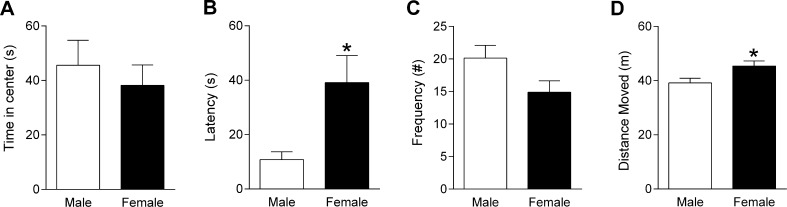
Females show enhanced anxiety-like behaviour and locomotor activity during open field testing. (A) There was no difference between males and females in the duration of time spent in the center of the open field. (B) Females showed an increased latency to first enter the center of the open field (^*^P < 0.05). (C) The frequency of entries into the center of the open field did not differ significantly between males and females. (D) The horizontal distance moved in the open field was increased in females (^*^P < 0.05).

It is possible that the sex differences in freezing in response to CS+ and CS− presentation during fear discrimination retrieval reported in Experiment 1A could have resulted from non-specific effects on anxiety-like behaviour and/or locomotor activity. To address this possibility we examined indices of these behaviours in males (n = 7) and females (n = 8) during open field testing in Experiment 1B. The time spent in, latency to enter, and frequency of entries into the center of the open field are shown in Fig. 2A–C. The two-way ANOVA for these anxiety-like behavioral measures revealed a significant main effect of measure (F_(2,26)_ = 5.66, P = 0.009) and a significant sex × measure interaction (F_(2,26)_ = 3.63, P = 0.041) but no main effect of sex (F_(1,16)_ = 1.82, P = 0.20). Post hoc analysis indicated that there was no difference between males and females in the time spent in the center (P > 0.05; Fig. 2A). However, females took significantly longer to enter the center, compared to males (P < 0.05; Fig. 2B). Females also made fewer entries into the center than males, although this did not reach statistical significance (P > 0.05; Fig. 2C). Locomotor activity in the open field is presented in Fig. 2D. Females showed a significant increase in the horizontal distance moved, compared to males (t_(13)_ = 2.45, P = 0.029; Fig. 2D). These results suggest that females displayed a subtle enhancement of anxiety-like behaviour and elevated locomotor activity in relation to males.

### 3.3 Experiment 2: Shock sensitivity does not differ between males and females

**Fig. 3:**
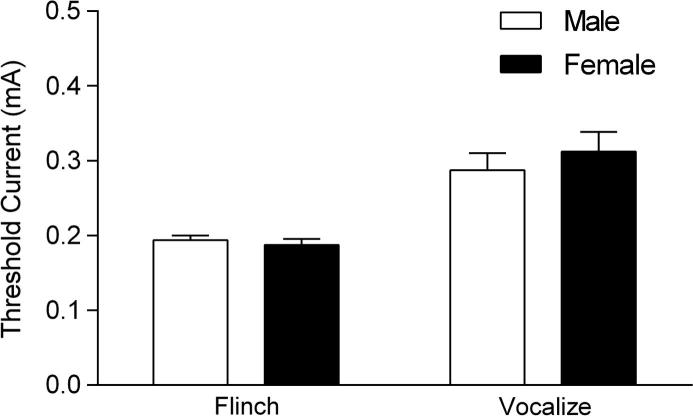
The threshold current eliciting flinch or vocalization responses does not differ between males and females, indicating a lack of sex differences in shock sensitivity.

The sex differences in fear discrimination retrieval reported in Experiment 1A could also have involved non-specific effects on nociception during fear discrimination training. To address this issue we examined shock sensitivity in separate cohorts of males (n = 8) and females (n = 8) in Experiment 2 (Fig. 3). The two-way ANOVA revealed a significant main effect of response type (F_(1,14)_ = 43.09, P < 0.0001) but no main effect of sex (F_(1,14)_ = 0.23, P = 0.64) or sex × response type interaction (F_(1,14)_ = 0.88, P = 0.36). These results indicate that there were no sex differences in shock sensitivity.

### 3.4 Experiment 3: Females show fear generalization with extended discrimination training due to impaired safety signaling

**Fig. 4:**
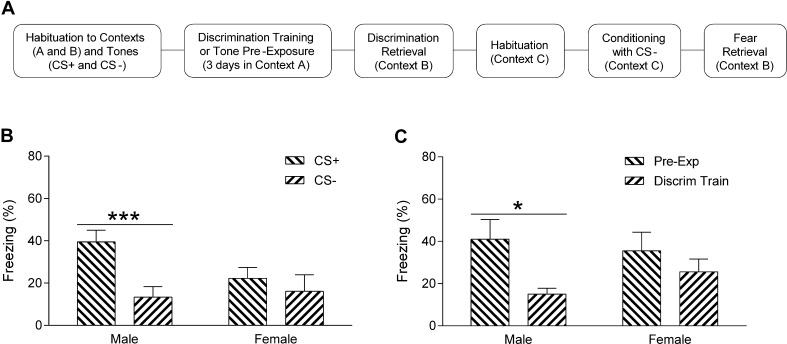
Sex differences in auditory fear discrimination with extended training involve altered safety signaling by the CS−. (A) Schematic representation of the discrimination and retardation testing paradigm used. (B) Freezing in response to the CS+ and CS− during discrimination retrieval testing after three days of training. Freezing was increased during CS+ compared to CS− presentation in males (^***^P < 0.001) but not females. (C) Males subjected to discrimination training (Discrim Train) followed by fear conditioning using the previous CS− as the cue showed decreased freezing to the cue during fear retrieval testing, compared to controls pre-exposed (Pre-Exp) to the cue before conditioning (^*^P < 0.05). There was no difference in freezing to the cue between females that underwent discrimination training and pre-exposed controls (note that the discrimination retrieval data in (B) is from the Discrim Train groups in (C).

The results from Experiment 1A indicated that males showed fear discrimination and females showed fear generalization after three days of discrimination training (Fig. 1D). To determine if this sex difference in fear discrimination with extended training involved altered safety signaling by the CS−, we subjected another cohort of rats to three days of discrimination training followed by retardation testing in Experiment 3 (Fig. 4A). Freezing in response to the CS+ and CS− during fear discrimination retrieval testing after three days of training is shown in Fig. 4B. The two-way ANOVA analysis revealed a significant main effect of CS type (F_(1,16)_ = 19.28, P = 0.0005) and a significant sex × CS type interaction (F_(1,16)_ = 7.43, P = 0.015) but no main effect of sex (F_(1,16)_ = 0.89, P = 0.36). Post hoc analysis indicated that males (n = 9) showed a significant increase in freezing during CS+ compared to CS− presentation (P < 0.001). In contrast, females (n = 9) showed no difference in freezing in response to the CS+ and CS− (P > 0.05). These results replicate our finding from Experiment 1A that males discriminated and females generalized between the CS+ and CS− after three days of fear discrimination training.

For the retardation test, after discrimination retrieval testing the same rats underwent fear conditioning using the CS− as the cue and fear retrieval was then tested (Fig. 4A). If later conditioning to the CS− is retarded, as indicated by a reduction in freezing during cue presentation at fear retrieval test, then this provides evidence that the CS− acquired the inhibitory properties of a safety cue during fear discrimination. Freezing in response to the cue during fear retrieval testing is presented in Fig. 4C. The two-way ANOVA analysis revealed a significant main effect of CS history (F_(1,34)_ = 5.77, P = 0.022) but no main effect of sex (F_(1,34)_ = 0.11, P = 0.74) or sex × CS history interaction (F_(1,34)_ = 1.15, P = 0.29). Despite there being no significant interaction, we were interested in examining differences in freezing between discrimination trained vs cue pre-exposed controls in males and females. Therefore direct comparisons were conducted using independent unpaired t-tests. Freezing was significantly decreased in response to the cue in males that had previously undergone discrimination training (n = 9), compared to controls (n = 10) pre-exposed to the cue before conditioning (t_(17)_ = 2.56, P = 0.021). In contrast, freezing during cue presentation in females previously subjected to fear discrimination (n = 9) did not differ from controls (n = 10) that underwent cue pre-exposure before conditioning (t_(17)_ = 0.91, P = 0.38). These results suggest that the CS− acted as a safety cue during fear discrimination in males but not females.

## 4 Discussion

This study investigated sex differences in auditory fear discrimination. In Experiment 1A we found that males showed marginal fear discrimination after limited training and successful discrimination with extended training. In contrast, females displayed fear discrimination with limited training and generalization after extended training. This indicates that sex differences in fear discrimination depended on the extent of training received. In Experiment 1B we found a subtle enhancement of anxiety-like behaviour and elevated locomotor activity in females during open field testing. In Experiment 2 we observed no sex differences in shock sensitivity. In Experiment 3 we again found that males showed fear discrimination while females showed fear generalization after extended training. We also provided evidence that the CS− signaled safety with extended fear discrimination training in males, whereas in females this safety signaling was impaired. These results confirm previous findings indicating sex differences in the inhibition of learned fear and extend them to the domain of fear discrimination involving auditory stimuli.

The finding that males receiving more fear discrimination training exhibited better discrimination performance agrees with previous studies showing a gradual improvement in discrimination over the course of extended training and is consistent with the idea that brief training paradigms lead to less stimulus specificity during fear learning (Antunes and Moita, 2010, Dunsmoor and Paz, 2015, Foilb and Christianson, 2016). Interestingly, we found opposing patterns of sex differences in fear discrimination with limited and extended training. In contrast to the improved discrimination with extended training that we observed in males, females did not discriminate between the CS+ and CS− after 2–3 days of training. This lack of discrimination was not due to a learning deficit because females clearly discriminated between the two cues after only one day of training. This suggests that sex differences in auditory perception are also unlikely to be involved, which is supported by previous studies showing that males and females did not differ in auditory appetitive discrimination (van Haaren and van Hest, 1989a, van Haaren and van Hest, 1989b). Our results instead suggest that females show fear discrimination after a single training session but that with repeated sessions they develop fear generalization. Previous studies investigating sex differences in aversive learning have shown faster acquisition of eyeblink conditioning and active avoidance in females that is most evident early on during learning (Dalla & Shors, 2009), which is similar to our results with limited discrimination training. It is unclear why fear generalization was observed after extended discrimination training in females but one possibility is that the stressful experience of the first day of training affected subsequent discrimination learning differently in males and females over the following day(s) of training. This idea is supported by the finding that acute stress the day before eyeblink conditioning improved learning in males but impaired learning in females (Wood & Shors, 1998). It is also in general agreement with other evidence indicating that stress and sex can interact to regulate learned fear and its inhibition through extinction (Baran et al., 2009, Keller et al., 2015). Recent evidence indicates sex differences in the social modulation of fear learning, where exposure to a conspecific subjected to fear conditioning affects subsequent fear learning (Mikosz, Nowak, Werka, & Knapska, 2015), suggesting that there may also be sex differences in the social modulation of fear discrimination.

It is worth noting that females and, to a lesser extent, males subjected to three days of discrimination training exhibited less freezing in response to the CS+ and CS− at discrimination retrieval test, compared to those that underwent one or two days of training. Although the reason for this remains unclear, one possibility is that the females in particular adopted less passive (i.e. freezing) and more active (i.e. escape-related) fear responding after extended discrimination training. This idea is supported by evidence from a recent study indicating that females were more likely to display active ‘darting’ movements as a type of fear response during auditory fear conditioning and its extinction, compared to males (Gruene, Flick, Stefano, Shea, & Shansky, 2015). Therefore future studies characterizing other fear responses apart from freezing in females and males during fear discrimination are warranted, especially given that Gruene et al. (2015) used a different rat strain than the one used in the present study.

We also found enhanced anxiety-like behaviour and locomotor activity in females but these results are unlikely to explain the sex differences that we observed in fear discrimination. Previous studies have reported decreased, unaltered or increased anxiety-like behaviour in females tested in the open field, whereas the finding of increased locomotion in females is more consistent across studies (Aguilar et al., 2003, Baran et al., 2010, Gray and Lalljee, 1974, Lehmann et al., 1999, Seliger, 1977). There are several possible reasons for this discrepancy between studies, including the measures quantified to index fear behaviour, the conditions under which testing occurred, and the strain used (Prut & Belzung, 2003). In our experimental setting we found no sex differences in the duration of time spent in or the frequency of entries into the center of the open field but we did find an increased latency to enter the center in females, despite the increase in locomotor activity that they also displayed, suggesting that females showed a subtle enhancement of anxiety-like behaviour. It could be argued that enhanced anxiety-like behaviour might contribute to fear generalization, which we observed in females after two or three days of discrimination training. However, this would not explain the fear discrimination that we observed in females after one day of training. Similarly, while increased locomotor activity might result in decreased freezing during presentation of both the CS+ and the CS− in females, it cannot explain the different patterns of fear discrimination observed with one or 2–3 days of training. Sex differences in nociception during fear discrimination training are also unlikely to account for our results as we found that males and females did not differ in their shock sensitivity. In contrast to the present findings, most previous studies have reported increased shock sensitivity in females (Dalla & Shors, 2009). Again, differences in the experimental conditions and the strain used between studies could account for this discrepancy.

We investigated the psychological process underlying sex differences in fear discrimination with extended training by determining if the CS− took on the inhibitory properties of a safety signal. Summation and retardation tests are used to demonstrate safety signaling by the CS− (Christianson et al., 2012, Sangha et al., 2013). During summation testing the CS+ and CS− are presented together and this reduces fear, compared to CS+ presentation alone, if the CS− acts as a safety signal. In our study it was not possible to use a summation test given that both cues were auditory stimuli. During retardation testing the CS− is used as the cue in subsequent fear conditioning. If the CS− signals safety then conditioning is retarded and fear in response to the cue at retrieval test is reduced in comparison to controls not subjected to prior discrimination training. We pre-exposed the controls to the same number of cues that were presented during fear discrimination to rule out the possibility that any differences in freezing during retrieval testing were attributable to an effect of latent inhibition, a form of learned irrelevance where cue pre-exposure impairs later conditioning to that cue (Young, Moran, & Joseph, 2005). This was also important to consider given that previous studies have shown sex differences in latent inhibition (Kaplan & Lubow, 2011). Compared to their respective controls, we found reduced fear in response to the prior safety cue in males but not females. This sex difference in the retardation test suggests that with extended discrimination training the CS− acted as a safety signal in males and that this safety signaling was impaired in females. An alternative interpretation is that females showed a deficit in latent inhibition rather than safety signaling. However, if latent inhibition was impaired then female controls might have been expected to show more fear at retrieval test compared to their male counterparts, which was not the case. Future studies examining sex differences in fear discrimination and safety signaling using cues from different sensory modalities, which would also allow for the assessment of summation testing, might prove useful in addressing this issue.

In fact, a previous study has examined sex differences and the role of estrogen in fear discrimination involving auditory and visual cues but there were also other important differences between that study and ours. Toufexis, Myers, Bowser, and Davis (2007) examined fear-potentiated startle in gonadectomized rats using an AX+/BX− discrimination paradigm, where presentation of A and X together predicted the US and B presented together with X signaled non-occurrence of the US. They also used a slow acquisition protocol, in which rats were subjected to fewer cue and US presentations over more days of training than in our study, to track changes in discrimination learning over time. Under these conditions both male and female shams showed fear discrimination over the course of training and during later retrieval testing. During summation testing, both male and female shams also exhibited less fear in response to the presentation of A and B together than when A was presented alone, providing evidence that B signaled safety. Furthermore, fear discrimination and safety signaling both depended on estrogen receptor signaling in females. Evidence indicates that sex differences in contextual fear discrimination also depend on estrogen (Lonsdorf et al., 2015, Lynch et al., 2013). A limitation of our study is that we did not account for variations in the estrous cycle phase of females, yet we still replicated our finding of fear generalization with extended training in a separate cohort of naturally cycling females. Moreover, a recent study in traumatized children found that girls showed impaired visual fear discrimination compared to boys (Gamwell et al., 2015), suggesting that sex differences in fear discrimination may involve the organizational effects of gonadal hormones during development and/or genetic factors that are independent of any hormonal effects. Nevertheless, when taken together with other evidence our results suggest that the generalized fear observed in intact females may have involved estrogen.

## 5 Conclusions

We found that females showed auditory fear discrimination with limited training and generalization with extended training due to impaired safety signaling. Our findings add to accumulating evidence indicating important sex differences in learned fear inhibition. From an adaptive perspective, there might be different circumstances which favor discrimination or generalization in relation to salient stimuli. Rapid discrimination between threat-related and harmless stimuli may conserve resources by restricting appropriate behavioral responding to a limited number of cues. On the other hand, generalizing across cues may enhance survival by promoting defensive responding to a wider range of stimuli that potentially predict threat, perhaps under more uncertain or stressful environmental conditions. However, when the balance tips too far towards generalization then this can lead to inappropriate fear in response to innocuous stimuli (Dunsmoor & Paz, 2015). Crucially, impaired fear discrimination and safety signaling are hallmark features of PTSD, which is also much more prevalent in women. Recent studies have begun to elucidate the neurobiological basis of sex differences in fear inhibition via extinction and further work is needed to determine if sex differences in fear discrimination involve similar, distinct or overlapping mechanisms.
